# Standardized approach for extubation during extracorporeal membrane oxygenation in severe acute respiratory distress syndrome: a prospective observational study

**DOI:** 10.1186/s13613-023-01185-y

**Published:** 2023-09-18

**Authors:** Roberto Roncon-Albuquerque, Sérgio Gaião, Francisco Vasques-Nóvoa, Carla Basílio, Ana Rita Ferreira, Alberto Touceda-Bravo, Rodrigo Pimentel, Ana Vaz, Sofia Silva, Guiomar Castro, Tiago Veiga, Hélio Martins, Francisco Dias, Catarina Pereira, Gonçalo Marto, Isabel Coimbra, Juan Ignacio Chico-Carballas, Paulo Figueiredo, José Artur Paiva

**Affiliations:** 1 Department of Emergency and Intensive Care Medicine, São João University Hospital Centre, Al. Prof. Hernâni Monteiro, 4200-319 Porto, Portugal; 2 UnIC@RISE, Department of Surgery and Physiology, Faculty of Medicine of the University of Porto, Porto, Portugal; 3 Department of Medicine, Faculty of Medicine, University of Porto, Porto, Portugal; 4 Department of Internal Medicine, São João University Hospital Centre, Porto, Portugal; 5 Critical Care Department, Hospital Álvaro Cunqueiro, Vigo (Pontevedra), Spain; 6 Department of Infectious Diseases, São João University Hospital Centre, Porto, Portugal

**Keywords:** Acute respiratory distress syndrome, Awake-ECMO, Extracorporeal membrane oxygenation, Extubation, Standardized approach, Weaning

## Abstract

**Background:**

Extubation during extracorporeal oxygenation (ECMO) in severe acute respiratory distress syndrome (ARDS) has not been well studied. Despite the potential benefits of this strategy, weaning from ECMO before liberation from invasive mechanical ventilation remains the most frequent approach. Our aim was to evaluate the safety and feasibility of a standardized approach for extubation during ECMO in patients with severe ARDS.

**Results:**

We conducted a prospective observational study to assess the safety and feasibility of a standardized approach for extubation during ECMO in severe ARDS among 254 adult patients across 4 intensive care units (ICU) from 2 tertiary ECMO centers over 6 years. This consisted of a daily assessment of clinical and gas exchange criteria based on an Extracorporeal Life Support Organization guideline, with extubation during ECMO after validation by a dedicated intensive care medicine specialist. Fifty-four (21%) patients were extubated during ECMO, 167 (66%) did not reach the clinical criteria, and in 33 (13%) patients, gas exchange precluded extubation during ECMO. At ECMO initiation, there were fewer extrapulmonary organ dysfunctions (lower SOFA score [OR, 0.88; 95% CI, 0.79–0.98; *P* = .02] with similar PaO_2_/FiO_2_) when compared with patients not extubated during ECMO. Extubation during ECMO associated with shorter duration of invasive mechanical ventilation (7 (4–18) vs. 32 (18–54) days; *P* < .01) and of ECMO (12 (7–25) vs. 19 (10–41) days; *P* = .01). This was accompanied by a lower incidence of hemorrhagic shock (2 vs. 11%; *P* = .05), but more cannula-associated deep vein thrombosis (49 vs. 31%; *P* = .02) and failed extubation (20 vs. 6%; *P* < .01). There were no increased major adverse events. Extubation during ECMO is associated with a lower risk of all-cause death, independently of measured confounding (adjusted logistic regression OR 0.23; 95% confidence interval 0.08–0.69, *P* = .008).

**Conclusions:**

A standardized approach was safe and feasible allowing extubation during ECMO in 21% of patients with severe ARDS, selecting patients who will have a shorter duration of invasive mechanical ventilation, ECMO course, and ICU stay, as well as fewer infectious complications, and high hospital survival.

**Supplementary Information:**

The online version contains supplementary material available at 10.1186/s13613-023-01185-y.

## Background

In patients with severe acute respiratory distress syndrome (ARDS) failing invasive mechanical ventilation (IMV) and rescued with extracorporeal membrane oxygenation (ECMO), the best weaning strategy remains unknown [1–3]. Prioritizing weaning from IMV may significantly reduce ventilator-induced lung injury and ventilator-associated pneumonia, as well as the complications of sedation and patient deconditioning [4]. However, deferring ECMO weaning could increase the risks of this invasive technique such as bleeding, thrombosis, hemolysis, and cannula-related infection [5–9]. Moreover, in awake patients with ECMO, specific risks such as accidental decannulation and ECMO equipment failure must also be considered [10]. In that context, the Extracorporeal Life Support Organization (ELSO) published a guideline for endotracheal extubation in patients with respiratory failure receiving ECMO, with clinical and gas exchange criteria to select patients to be safely and successfully managed without IMV [11].

Despite the potential benefits of early liberation from IMV in severe ARDS, weaning from ECMO before liberation from IMV remains the most frequent approach [1]. In a recent survey that included 253 ECMO centers worldwide, only one-third reported performing extubation during ECMO [2]. This latter strategy was considered mostly for other causes of respiratory failure such as end-stage respiratory disease awaiting lung transplantation [2], where there is evidence of the feasibility of an awake non-intubated approach during ECMO [12–15]. Differently, spontaneous breathing in severe ARDS could be more challenging due to spontaneous hyperventilation and strenuous respiratory muscle effort [4], lung derecruitment and collapse delirium, inability to handle pulmonary secretion [16], hemodynamic instability, and multiple organ failure [2].

To assess the safety and feasibility of a standardized approach for extubation during ECMO in severe ARDS, we conducted a prospective observational study among adult patients across 4 ICUs from 2 tertiary ECMO centers over 6 years. This consisted of a daily assessment of clinical and gas exchange criteria based on an Extracorporeal Life Support Organization guideline [11], with extubation during ECMO after validation by a dedicated intensive care medicine specialist.

## Methods

A prospective observational study of patients with severe ARDS rescued with ECMO in 2 European tertiary ECMO centers from January 2016 to July 2022 was carried out. The institutional Ethics Committee reviewed and approved the study and waived the requirement for patient consent (Approval number: CES 205/2022). All the procedures followed in the study were by the ethical standards of the institutional responsible committee on human experimentation and with the Helsinki Declaration of 1975, as most recently amended.

ECMO case volume, clinical criteria for ECMO consideration, and ECMO technical considerations are described in detail in the supplemental data. The initial type of ECMO support was venovenous (VV), except for one trauma patient in the CTRL group with both lung and cardiac contusion and simultaneous severe respiratory failure and cardiogenic shock, in which venovenoarterial (VVA) ECMO was the initial type of ECMO support. The 2 centers have intensivist-led respiratory ECMO programs comprising dedicated intensive care medicine specialists that are responsible for: (i) the decision to initiate ECMO; (ii) coordination of ECMO patient retrieval from referring hospitals; (iii) performing percutaneous ECMO cannulation; (iv) daily monitoring and management of ECMO-related complications (e.g., circuit exchange for ECMO-related hemolysis); (v) the decision to wean from ECMO, and (vi) to perform ECMO decannulation. A team of ECMO Specialists on-call 24/7 is responsible for (i) ECMO circuit priming; (ii) daily circuit maintenance (e.g., transmembrane pressure monitoring); (iii) ECMO circuit component exchange (e.g., oxygenator exchange), and (iv) ECMO circuit management during patient retrieval from referring hospitals. The nurse-to-ECMO patient ratio was 1:1 and was not modified in case of extubation during ECMO support.

### A standardized approach for extubation during extracorporeal membrane oxygenation

**Fig. 1:**
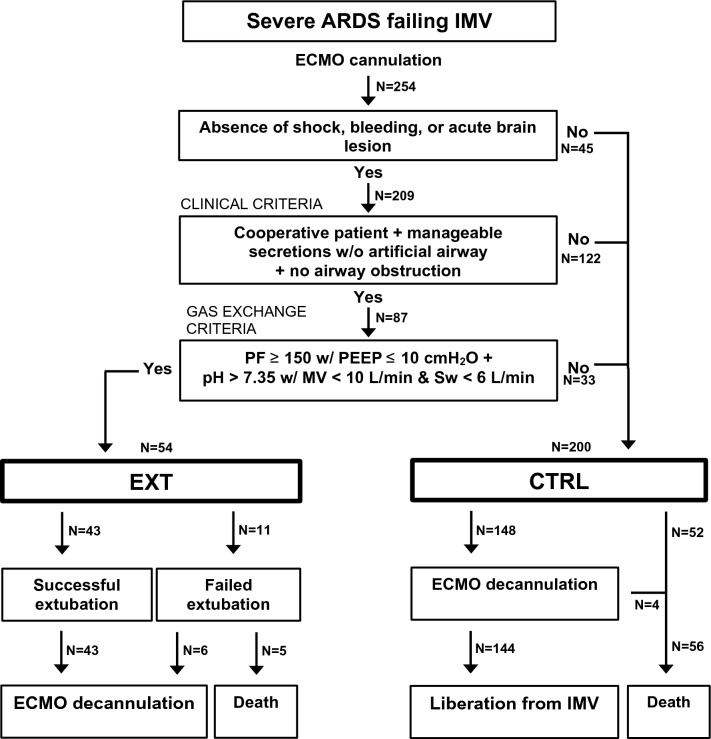
Flowchart for extubation during extracorporeal membrane oxygenation (ECMO) in severe acute respiratory distress syndrome (ARDS). Following initial patient stabilization and optimization of ECMO support, conscious sedation was pursued in the absence of circulatory shock, significant bleeding, or acute brain injury. A daily assessment of clinical and gas exchange criteria based on an Extracorporeal Life Support Organization guideline [11] was then performed, with extubation during ECMO after validation by a dedicated intensive care medicine specialist. CTRL, group without extubation during ECMO; EXT, group with extubation during ECMO; IMV, invasive mechanical ventilation; MV, minute ventilation; PEEP, positive end-expiratory pressure; PF, the ratio between the partial pressure of oxygen in arterial blood (PaO_2_; mmHg) and the fraction of inspired oxygen (FiO_2_; %); Sw, ECMO sweep gas flow

After cannulation, a standardized approach for extubation during ECMO was performed (Fig. 1). Given that ECMO consideration required sustained clinical deterioration despite optimal conventional treatment, including prone position unless contraindicated, prone positioning was not performed routinely after ECMO implantation. Following initial patient stabilization and optimization of ECMO support, weaning from neuromuscular blocking agents (NMB) and sedation was pursued to achieve conscious sedation (Richmond Agitation-Sedation Scale (RASS) =  − 1/0) if all the following were absent: (i) circulatory shock; (ii) significant bleeding, and (iii) acute brain injury. Conversion to VVA ECMO during VV ECMO support was considered for circulatory support in the setting of cardiovascular collapse complicating persistent severe respiratory failure. There were no cases of venoarterial (VA) ECMO, namely as initial ECMO circuit configuration in the setting of respiratory failure with associated septic shock, or as conversion from VV ECMO.

Whenever conscious sedation was successfully achieved, a daily assessment of clinical and gas exchange criteria based on the Extracorporeal Life Support Organization (ELSO) guideline for ‘Endotracheal extubation in patients with respiratory failure receiving venovenous ECMO’ [11] was performed. Readiness for endotracheal extubation was considered when: (i) the patient was cooperative enough not to be at significant risk for dislodgement of cannulas or other important catheters or devices; (ii) secretions were manageable without an artificial airway, and (iii) there was no airway obstruction. Regarding the gas exchange criteria, the following parameters had to be present: (i) the ratio of the PaO_2_ (arterial oxygen partial pressure obtained from an arterial blood gas) to the FiO_2_ (fraction of inspired oxygen expressed as a decimal) (PF ratio) ≥ 150 with positive end-expiratory pressure (PEEP) ≤ 10 cmH_2_O, and (ii) pH > 7.35 with minute ventilation (MV) < 10 L/min under pressure support ventilation while receiving a sweep gas flow < 6 L/min. These criteria differed from the ELSO guideline [11] for the P/F ratio (≥ 150 vs. ≥ 200 in the ELSO guideline) and for the PEEP value (≤ 10 vs. ≤ 5 cmH_2_O in the ELSO guideline). Whenever the predefined clinical and gas exchange criteria were fulfilled, validation by a dedicated intensive care medicine specialist from the ECMO Program was required before the final decision for extubation during ECMO was made.

After extubation during ECMO (EXT group) high-flow nasal cannula was initiated immediately after extubation with flow started at 40 L/min and FiO_2_ set to peripheral capillary oxygen saturation (SpO_2_) > 92%. Sweep gas flow was then titrated to avoid respiratory acidosis and to minimize the work of breathing, with a respiratory rate goal of 10–20 breaths per minute. Paracetamol was used for analgesia; whenever necessary, opioids (morphine or fentanyl), titrated to the lowest effective dose, were also administered. Dexmedetomidine was used for anxiolysis; whenever necessary, benzodiazepines, titrated to the lowest effective dose, were also administered. Fever control was pursued using non-steroidal anti-inflammatory drugs and/or metamizole unless contraindicated. Whenever present, the non-productive cough was suppressed with codeine and titrated to the lowest effective dose.

No specific standardized criteria were used for reintubation in the EXT group. Whenever a significant patient deterioration occurred after extubation during ECMO support, a dedicated intensive care medicine specialist from the ECMO Program was involved to exclude a lack of ECMO support optimization, being part of the decision to reintubate when indicated. In this group, tracheostomy was considered after failed extubation.

When significant improvement in native lung function was suspected (increased PF ratio with better lung aeration evaluated by chest X-ray or lung ultrasound), ECMO blood flow was stepwise reduced to 3.0 L/min. Sweep gas flow was then tapered to 1 L/min with close monitoring of respiratory rate and effort. After a 12–24 h period of clinical stability, the sweep gas flow was finally shut off for > 30 min. If blood gases remained stable (PF ratio ≥ 150, no respiratory effort, and absence of uncompensated respiratory acidosis) the ECMO system was removed.

When the clinical and gas exchange criteria for extubation during ECMO were not met at the time of consistent native lung function improvement (increased PF ratio, improved respiratory system (RS) compliance, and better lung aeration evaluated by chest X-ray or lung ultrasound), weaning from ECMO was prioritized (CTRL group). Invasive mechanical ventilation parameters were then progressively reduced to FiO_2_ ≤ 0.5, PEEP ≤ 10 cmH_2_O, and plateau pressure (PPlat) ≤ 25 cmH_2_O. ECMO blood flow was then stepwise reduced to 3.0 L/min. Thereafter sweep gas flow was tapered and finally shut off for > 30 min. If blood gases remained stable (PF ratio ≥ 150, MV < 10 L/min with no respiratory effort, and absence of uncompensated respiratory acidosis), the ECMO system was removed. In this group, tracheostomy was considered when no significant native lung function improvement was observed during the second week of ECMO support.

### Data collection and statistical analysis

Statistical analysis methods used in the study are detailed in the supplemental data.

## Results

### Flowchart for extubation during ECMO in severe ARDS

Of the 254 patients with severe ARDS included in the study (Fig. 1), 54 (21%) met the clinical and gas exchange criteria and were extubated during ECMO (EXT group). Of the remaining 200 patients (CTRL group) 45 (18%) patients had a circulatory shock, significant bleeding, or acute brain injury contraindicating conscious sedation, 122 (48%) patients did not reach the clinical criteria for extubation during ECMO, and 33 (13%) patients fulfilling the clinical criteria did not meet the gas exchange criteria for extubation during ECMO. Conversion to VVA ECMO was performed for circulatory support during VV ECMO in 5 patients due to: (i) acute cor pulmonale in 4 patients; (ii) cardiac tamponade complicating ECMO cannulation in one patient. All but one patient were from the CTRL group. In the patient from the EXT group, conversion to VVA ECMO was performed in the setting of acute cor pulmonale after reintubation.

Eleven patients (20%) failed extubation in the EXT group. The reasons for reintubation were: (i) excess pulmonary secretions in 4 patients; (ii) respiratory muscle fatigue in 3 patients; (iii) upper airway obstruction in 2 patients; (iv) refractory hypoxemia in 1 patient; and (v) cardiac arrest in 1 patient. Of the 11 patients with failed extubation 5 died, while all patients successfully extubated in the EXT group survived hospital discharge. In the CTRL group, ECMO weaning and decannulation were achieved in 148 patients (74%), while 52 (26%) patients died during ECMO. Of the 148 patients with ECMO decannulation in the CTRL group, 144 (97%) were liberated from IMV and survived to hospital discharge.

### Baseline patient characteristics

Patients included in the study were relatively young and mostly male. Cardiovascular risk factors were common, with hypertension more frequently present in the CTRL group (Table 1).

**Table 1:** Baseline characteristics

Characteristics	All (*n* = 254)	CTRL (*n* = 200)	EXT (*n* = 54)	*P*-value
Age (years)	51 (42–59)	52 (42–60)	50 (41–58)	0.29
Male gender	179 (70)	141 (70)	38 (70)	0.98
Comorbidities				
Hypertension	95 (37)	82 (41)	13 (24)	0.02
Obesity	91 (36)	72 (36)	19 (35)	0.91
Dyslipidemia	86 (34)	70 (35)	16 (30)	0.46
Smoking	67 (26)	54 (27)	13 (24)	0.66
Diabetes mellitus	42 (16)	35 (18)	7 (13)	0.43
Alcoholism	32 (13)	29 (14)	3 (6)	0.08
Type of ARDS				
Pulmonary	227 (90)	176 (89)	51 (94)	0.23
Extrapulmonary	25 (10)	22 (11)	3 (6)	
ARDS etiology				
Viral pneumonia	117 (46)	91 (46)	26 (48)	0.78
COVID-19	82 (32)	64 (32)	18 (33)	0.85
Bacterial pneumonia	37 (15)	28 (14)	9 (17)	0.62
Pneumonia without SPD	36 (14)	28 (15)	8 (15)	0.88
Lung contusion	25 (10)	19 (10)	6 (11)	0.72
Extrapulmonary sepsis	14 (6)	12 (6)	2 (4)	0.74
Other	25 (10)	22 (11)	3 (6)	0.23
Pre-ECMO course (days)				
Hospital to ECMO	4.0 (1.0–8.0)	4.0 (1.0–8.0)	4.0 (1.0–7.0)	0.33
IMV to ECMO	2.5 (1.0–6.0)	3.0 (1.0–6.0)	2.0 (1.0–5.0)	0.07
Pre-ECMO management				
NMB	245 (98)	193 (97)	52 (98)	1.00
Prone position	198 (79)	154 (78)	44 (83)	0.41
ECMO retrieval	173 (68)	134 (67)	39 (72)	0.46
SAPS II	46 (34–57)	49 (36–60)	39 (24–54)	< 0.01
SOFA	8.0 (5.0–11)	8.0 (6.0–12)	7.0 (4.0–9.0)	< 0.01
Murray score	3.3 (3.0–3.7)	3.3 (3.0–3.7)	3.3 (3.0–3.5)	0.21
RESP score	3.0 (1.0–5.0)	3.0 (1.0–5.0)	4.0 (1.0–5.0)	0.12

Data are presented as numbers (%) or median (interquartile range)

*COVID-19* COVID-19-related pneumonia, *CTRL* group without extubation during ECMO, *EXT* group with extubation during ECMO, *NMB* neuromuscular blocking agents, *RESP Score* Respiratory Extracorporeal Membrane Oxygenation Survival Prediction score, *SAPS II* Simplified Acute Physiology Score II at ICU admission, *SOFA* Sequential Organ Failure Assessment score at ECMO cannulation, *SPD* specific pathogen detected; SOFA score was calculated in the last day before ECMO implantation

Most patients (90%) had a pulmonary type of ARDS, with viral pneumonia in 117 (46%) patients (82 (32%) of which had COVID-19-related pneumonia), bacterial pneumonia in 37 (15%) patients, and pneumonia without a specific pathogen detected in 36 (14%). Lung contusion was the cause of ARDS in 25 (10%) patients, 14 (6%) had extrapulmonary sepsis, while 25 (10%) patients had an ARDS of other etiology. No significant differences were detected in the ARDS type or etiology between groups.

Regarding the pre-ECMO course, no significant differences were detected between groups in the time from hospital admission to ECMO initiation or in the duration of IMV before ECMO cannulation. Regarding pre-ECMO management, the rate of NMB use and prone position was high (98 and 79%, respectively) and did not differ between groups.

Most patients (68%) were retrieved from referring hospitals, with no significant differences between groups. EXT group had lower SAPS II and lower SOFA scores, but similar Murray scores. Hospital survival predicted by the Respiratory Extracorporeal Membrane Oxygenation Survival Prediction score (RESP score) was 76%, being similar in EXT and CTRL groups.

### Ventilatory parameters and gas exchange before and during initial ECMO support

Pre-ECMO ventilator parameters and respiratory mechanics differed between groups: EXT group presented lower FiO_2_ and higher static RS compliance when compared with the CTRL group, with no significant differences in PEEP, tidal volume, minute ventilation, and plateau pressure (Additional file 1: Table S1). After ECMO initiation (ECMO Day 1) FiO_2_ was still lower in the EXT group, but with no significant differences in the other ventilatory parameters. On the day of liberation from IMV, the EXT group presented higher PEEP, but lower tidal volume, minute ventilation, and static RS compliance, when compared with CTRL.

No significant differences in gas exchange parameters such as PF ratio, partial pressure of carbon dioxide in arterial blood (PaCO_2_), and pH between EXT and CTRL groups pre-ECMO and at ECMO Day 1 (Additional file 1: Table S1). On the day of ECMO implantation, arterial blood lactate was slightly higher in the CTRL group when compared with the EXT group. Regarding ECMO support parameters, ECMO blood flow and sweep gas flow also did not differ between groups on ECMO Day 1. ECMO blood flow also did not differ between groups on ECMO day 3 (CTRL group: 4.3 (3.9–4.6) vs. EXT group: 4.2 (3.9–4.5) L/min; *P* = 0.28) and on ECMO Day 7 (CTRL group: 4.2 (3.8–4.7) vs. EXT group: 4.2 (3.8–4.7) L/min; *P* = 0.89). On the day of liberation from IMV, the EXT group presented a lower PF ratio and pH, and higher PaCO_2_ when compared with the CTRL group (Additional file 1: Table S2). In the EXT group, ECMO blood flow and sweep gas flow were lower on the day of decannulation when compared to the day of extubation. ECMO blood flow and sweep gas flow were also lower on the day of decannulation in the EXT group when compared to the CTRL group (Additional file 1: Table S2).

Given the significant differences observed between EXT and CTRL groups in some pre-ECMO characteristics, a multivariate analysis was performed. Arterial hypertension, pre-ECMO FiO_2_, lactate, and SAPS II score were identified as relevant predictors of group (EXT vs. CTRL) assignment (Table 2).

**Table 2:** Logistic regression model including pre-ECMO variables describing independent associations with group assignment

Variables	OR (95% CI)	*P*-value
Lactate (log_2_-transformed)	0.44 (0.26–0.74)	0.002
Hypertension	0.46 (0.22–0.97)	0.041
FiO_2_	0.98 (0.96–0.99)	0.035
SAPS II	0.97 (0.95–0.99)	0.004

Results are presented as odds ratio (OR) with correspondent 95% confidence interval. CTRL, group without extubation during ECMO; EXT, group with extubation during ECMO; SAPS II, Simplified Acute Physiology Score II at ICU admission; FiO_2_, fraction on inspired oxygen

### ECMO-associated complications and major adverse events

The incidence of nosocomial infections was high (68%), and significantly more frequent in the CTRL group (Table 3). Ventilator-associated pneumonia was the most frequent nosocomial infection, followed by the bloodstream, urinary tract, abdominal, and skin and soft-tissue infections, respectively. The incidence of ventilator-associated pneumonia was higher in the CTRL group. Although this group also presented a higher incidence of bloodstream, urinary tract, abdominal and skin, and soft-tissue infections, it did not reach statistical significance. The lower incidence of nosocomial infection observed in the EXT group was accompanied by a shorter duration of antibiotic therapy in the ICU and more antibiotic-free days in the ICU (%).

**Table 3:** ECMO-associated complications and major adverse events

	All (*n* = 254)	CTRL (*n* = 200)	EXT (*n* = 54)	*P*-value
Infectious complications				
Nosocomial infections	172 (68)	147 (74)	25 (46)	< 0.01
Ventilator-associated pneumonia	147 (58)	127 (64)	20 (37)	< 0.01
Bloodstream infection	59 (23)	51 (26)	8 (15)	0.10
Urinary tract infection	43 (17)	37 (18)	6 (11)	0.20
Abdominal infection	9 (4)	9 (5)	0 (0)	0.21
Skin and soft-tissue infection	8 (3)	8 (4)	0 (0)	0.21
Duration of antibiotic therapy in ICU (days)	16 (9–35)	20 (11–41)	7 (6–14)	< 0.01
Antibiotic-free days in ICU (%)	35 (10–52)	29 (9–50)	42 (25–62)	0.01
Bleeding complications				
Hemorrhagic shock	22 (8.7)	21 (10.6)	1 (1.9)	0.05
Intracerebral hemorrhage	5 (2.0)	3 (1.5)	2 (3.7)	0.29
Thrombotic complications				
Cannula-associated deep vein thrombosis	69 (36)	45 (31)	24 (49)	0.02
Limb ischemia	3 (1.2)	3 (1.5)	0 (0)	0.49
Ischemic stroke	1 (0.4)	1 (0.5)	0 (0)	1.00
Major adverse events				
Refractory hypoxemia	15 (5.9)	12 (6.0)	3 (5.6)	1.00
Cardiac arrest	8 (3.2)	5 (2.5)	3 (5.6)	0.37
Accidental decannulation	1 (0.4)	0 (0)	1 (1.9)	0.21

Data are presented as numbers of cases (percentage). CTRL, group without extubation during ECMO; ECMO, extracorporeal membrane oxygenation; EXT, group with extubation during ECMO

Hemorrhagic shock was found in 11% of the CTRL group, with one case in the EXT group due to a non-fatal accidental ECMO decannulation of a 19-Fr and 15-cm-long jugular return cannula. This adverse event occurred 5 days after a failed extubation attempt when the patient was with IMV under light sedation. This was immediately handled with ECMO circuit clamping and local compression, conversion to femoro-femoral veno-venous ECMO configuration, and blood transfusion. The incidence of intracerebral hemorrhage was low (2.0%) and did not differ between groups.

Cannula-associated deep vein thrombosis was more frequent in the EXT group. No significant differences were found in the incidence of limb ischemia and ischemic stroke between groups. Before ECMO implantation the EXT group presented higher platelet counts and shorter aPTT and PT values, with no significant differences in D-dimers and fibrinogen levels, when compared to the CTRL group (Additional file 1: Table S3). During ECMO support (ECMO Day 1, 3, and 7) higher platelet counts were also consistently observed in the EXT group, while no significant differences in aPTT, PT, d-dimers, and fibrinogen levels were detected between groups.

Regarding major adverse events, the incidence of refractory hypoxemia, cardiac arrest, and accidental decannulation was low and did not significantly differ between groups.

### Clinical outcomes

EXT group presented shorter ECMO runs, IMV duration, and ICU length of stay, even after adjustment for clinically relevant covariates (Table 4; Additional file 1: Table S5). In this group, the delay between successful extubation and ECMO decannulation was 6 (4–10) days. Failed extubation rate was lower in the CTRL group, but the tracheostomy rate was higher. In this group, tracheostomy was performed mostly before ECMO weaning (78 vs. 22%), 21 (16–31) days after IMV initiation, and 17 (11–25) days after ECMO implantation. In the EXT group, a tracheostomy was performed after extubation failure, except for one case in which a second extubation attempt was made. Regarding the health-related quality of life evaluated 3–6 months after hospital discharge, the EQ-5D-5L level summary score was significantly lower in the EXT group, while no significant differences were detected in the EQ-VAS score between groups. All-cause mortality at 150-day follow-up was lower in EXT (Table 4).

**Table 4:** Clinical outcomes

	All (*n* = 254)	CTRL (*n* = 200)	EXT (*n* = 54)	*P*-value
ECMO duration (days)	17 (10–35)	19 (10–41)	12 (7–25)	0.01
IMV duration (days)	25 (13–46)	32 (18–54)	7 (4–18)	< 0.01
Failed extubation (%)	24 (9.5)	13 (6.5)	11 (20.4)	< 0.01
Tracheostomy (%)	123 (48.6)	112 (56.3)	11 (20.4)	< 0.01
ICU-LOS (days)	32 (18–56)	37 (22–63)	19 (13–32)	< 0.01
Hospital survival (%)	193 (76.0)	144 (72.0)	49 (90.7)	< 0.01
EQ-5D-5L	9 (7–11)	10 (9–12)	8 (7–10)	0.03
EQ-VAS	75 (60–80)	75 (60–80)	75 (60–85)	0.63

Data are presented as numbers of cases (%) or median (interquartile range)

*CTRL* group without extubation during ECMO, *EXT* group with extubation during ECMO, *EQ-5D-5L* EuroQol-5 dimension self-assessed, health-related, quality of life questionnaire presented as a level summary score, *EQ-VAS* EuroQol vertical visual analog scale for self-rated health, *ICU* intensive care unit, *LOS* length of stay

**Fig. 2:**
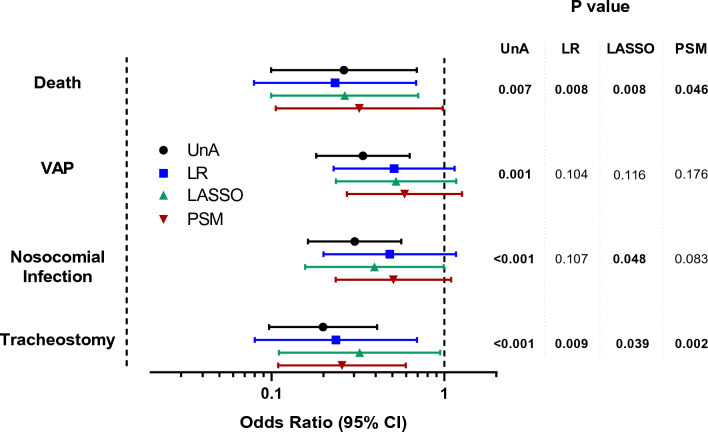
Unadjusted (UnA) and adjusted study of clinical outcomes. Standard logistic regression (LR), least absolute shrinkage and selection operator (LASSO) logistic regression and logistic regression after propensity-score matching (PSM) were used to assess clinical outcomes in EXT group with CTRL group as reference, presented as odds ratio with their respective 95% confidence intervals. VAP, ventilator-associated pneumonia. Corresponding P-values are given on the right

The impact of EXT on key clinical outcomes was assessed using multiple approaches to control for confounding, namely standard unadjusted and adjusted logistic regression, least absolute shrinkage, and selection operator (LASSO) logistic regression and propensity-score (PS) matching followed by standard logistic regression (Fig. 2). After PS matching (54 pairs), groups were well balanced for all variables included in the PS model, as appraised by absolute standardized bias < 10% (Additional file 1: Table S4).

EXT group was associated with a lower risk of 150-day all-cause mortality (OR 0.23–0.32; p < 0.05), independently of the key clinical covariates and confounding adjustment method (Fig. 2; Additional file 1: Fig. S1). EXT group associated with lower risk of nosocomial infection and VAP in the unadjusted analysis. However, these associations were highly dependent on the ICU length of stay, which was shorter in the EXT group (Table 4) and lost their statistical significance in the adjusted models (Fig. 2). Notably, EXT group was also associated with decreased risk of tracheostomy, independently of the adjustment method (Fig. 2).

## Discussion

In the present study, the safety and feasibility of a standardized approach for extubation during ECMO in severe ARDS were evaluated. This consisted of a daily assessment of clinical and gas exchange criteria based on an Extracorporeal Life Support Organization guideline [11], with extubation during ECMO after validation by a dedicated intensive care medicine specialist. Using this approach, 21% of patients with severe ARDS could be extubated during ECMO support.

To evaluate the safety of our standardized approach for extubation during ECMO in severe ARDS, we analyzed ECMO-related complications, major adverse events, nosocomial infections in the ICU, and main clinical outcomes. Extubation during ECMO did not associate with an increased incidence of major adverse events. This could be related, at least in part, to the fact that extubation during ECMO was associated with shorter ECMO runs, a well-established determinant of ECMO-related complications [17]. Of note, one case of non-fatal accidental ECMO decannulation occurred in the EXT group, 5 days after a failed extubation attempt, when the patient was on IMV under light sedation. An accidental decannulation requiring emergent intubation and brief cardiopulmonary resuscitation was also reported in a case series on the use of ECMO instead of IMV in ARDS by Hoeper et al. [10]. This underscores the importance of providing ECMO in centers with extensive experience for the immediate correct management of ECMO-related complications [18, 19]. Failed extubation rate was higher in the EXT group when compared with the CTRL group. Objective criteria for patient extubation under ECMO support are not clearly established and are based on expert recommendations from experienced centers [1, 2, 11, 20], and further studies are warranted to better define patient selection for this weaning strategy in severe ARDS. A higher incidence of cannula-associated deep vein thrombosis was observed in the EXT group. During ECMO support, higher platelets with similar systemic anticoagulation levels (as evaluated by the aPTT) could eventually have contributed, at least in part, to the higher rate of cannula-associated deep vein thrombosis observed in this group. In a recent experimental study, the reduction of platelets within an in vitro ECMO test circuit was associated with lower clot stability [21]. We did not collect individual patient data on the cannulae caliber, so we cannot hypothesize if this could have contributed to the observed differences in deep vein thrombosis between groups. Regarding ECMO blood flow, it did not differ between groups during the first week of ECMO support, so it is unlikely that it has contributed to the observed differences in cannula-associated deep vein thrombosis.

Despite the low level of systemic anticoagulation used in our study, the frequency of hemorrhagic shock in the CTRL group goes in line with the results of a systematic review and meta-analysis of complications and mortality of VV ECMO for refractory acute respiratory distress syndrome [5], in which significant bleeding was reported in 10.4% of cases. In a recent study, longer duration of ECMO support and lower platelet count were identified as independent risk factors for hemorrhage in adults on ECMO [22]. Both factors were observed in the CTRL group when compared with EXT group.

During ECMO support nosocomial infections, especially ventilator-associated pneumonia, are common and associated with worse outcomes [23, 24]. Extubation during ECMO was associated with a lower incidence of nosocomial infections in the ICU, namely ventilator-associated pneumonia, with a corresponding shorter duration of antibiotic therapy and more antibiotic-free days in the ICU (%). This is likely related to the shorter IMV and ICU length of stay in this group, which are two main determinants of ICU-acquired infections [25, 26]. In two studies analyzing the feasibility of patient extubation during ECMO, mostly for cardiac support, a lower incidence of ventilator-associated pneumonia was also observed [27, 28]. Regarding patient outcome, extubation during ECMO was associated with higher hospital survival when compared with the CTRL group, which could further indicate the safety of our standardized approach for extubation during ECMO in severe ARDS.

Patients in the CTRL group presented higher SAPS II and SOFA scores at ECMO initiation, with no significant differences in PF ratio and Murray score, indicating more extrapulmonary organ dysfunctions when compared to the EXT group. This agrees with the ELSO guideline on extubation during ECMO in patients with respiratory failure [11] and the initial published experience [4, 10, 29], in which readiness for extubation depends on the absence of circulatory shock or multi-organ failure, as well as on whether the patient is awake and cooperative.

Although patients extubated during ECMO had higher baseline static RS compliance, this was no longer apparent after ECMO initiation and adjustment of IMV to lower tidal volumes. Importantly, in our study, ECMO support allowed stable and adequate arterial blood gases for extubation, even in the face of significantly reduced RS compliance and extensive bilateral pulmonary infiltrates, typically observed in severe ARDS [30]. Recently, in a case report, Schmidt et al. were able to successfully treat COVID-19-associated severe ARDS with prolonged (41 days) ‘awake ECMO’ [31]. Before ECMO cannulation the chest X-ray showed extensive bilateral pulmonary infiltrates, with the chest computed tomography, performed on Day 20, still presenting extensive bilateral parenchymatous condensations. Analogously, extensive bilateral pulmonary infiltrates did not preclude successful extubation before ECMO decannulation of 5 patients with COVID-19-associated severe ARDS [32]. Differences in RS compliance between CTRL and EXT groups on the day of liberation from IMV could not be explained solely by the reduction of ventilation during ECMO, being also affected by the different timing of extubation (7 (4–18) vs. 32 (18–54); *P* < 0.01), meaning probably a different ARDS phase. Moreover, the difficulty in measuring RS compliance during assisted breathing [33, 34] should also be considered when interpreting these observed differences.

In our study, high-flow nasal oxygen was routinely used immediately after extubation during ECMO, with noninvasive ventilation being restricted to selected patients with obesity or COPD. This was well tolerated, importantly contributing to the success of this weaning strategy. High-flow nasal oxygen was also the preferred (66%) oxygen therapy modality for extubated ECMO patients in a retrospective study of 12 consecutive severe ARDS patients supported with ECMO [35]. Differently, in a previous study by Crotti et al. [16] spontaneously breathing in early ARDS on ECMO (mean duration of IMV was 3 days) was found to be very difficult to manage, with high levels of PEEP through CPAP required to limit lung derecruitment and collapse. This was attributed to the huge degree of lung inflammation, parenchymal edema, and consequent alveolar collapse, with other organ dysfunctions and septic shock often complicating the clinical picture. In our study, spontaneous breathing was implemented at a later stage of ARDS (mean duration of IMV was 7 days) and in the absence of significant extrapulmonary organ dysfunctions, which could have contributed to favorable clinical outcomes. However, considering the recently published evidence in patients at high risk of extubation failure [36], as well as the median PEEP value at the time of extubation (8 cmH_2_O) in the EXT group, a routine strategy of cycles of noninvasive ventilation or CPAP with a high-flow nasal cannula, to avoid possible derecruitment after extubation (unless there are contraindications or the patient does not tolerate it), could have been of value to reduce extubation failure.

Health-related quality of life evaluated 3–6 months after hospital discharge, as evaluated by the EQ-5D-5L instrument, was significantly improved in the EXT group when compared with the CTRL group, suggesting that the weaning strategy of extubation during ECMO support in severe ARDS could associate with improved patient recovery. However, further research is needed to clarify the clinical significance of these results, given that in our study no differences were detected between groups at follow-up in patient self-rated health (as evaluated by the EQ-VAS), the sample size is relatively small, there is no data on deconditioning such as limb muscle strength [37], and there is no information regarding delirium/delirium screening, all considered relevant for the potential benefits of 'awake ECMO [4].

This study has several limitations that should be addressed. It is a two-center study over 6 years, which could limit its internal and external validity. The studied groups (EXT and CTRL groups) are not well-balanced regarding baseline patient characteristics, which may have affected the group assignment and the clinical outcomes. The observational design of the study does not infer causality, namely, it does not allow us to distinguish the impact on the clinical outcomes (e.g., mortality and nosocomial infections) of meeting the clinical and gas exchange criteria or being extubated during ECMO. Moreover, given that during the ECMO run the gas exchanges depend on the patient’s interactions with the extracorporeal support, it would have been necessary to measure oxygen consumption (VO_2_) and CO_2_ removal (VCO_2_) by the ventilator and by the ECMO circuit to understand the relative contribution of the native and the membrane lung to the gas exchanges during extubation. We also did not collect individual patient data on the timing of the first assessment for extubation after ECMO cannulation, which could have provided further information on the usual management (such as prone positioning, neuromuscular blocker agents, and sedation) after ECMO implantation. Finally, our study did not evaluate the role of ECMO initiation in ARDS patients before endotracheal intubation. Although it has been previously shown to be feasible [10, 29, 38], a recent study on 18 adults patients with COVID-19-associated severe ARDS from 4 German tertiary care ICUs did not recommend this ‘awake-ECMO’ approach, as a high rate of patients receiving ‘awake-ECMO’ were finally intubated (78%) and those subsequently intubated seem to have higher mortality than patients managed conventionally with IMV and ECMO [39].

## Conclusions

A standardized approach was safe and feasible allowing extubation during ECMO in 21% of patients with severe ARDS, selecting patients who will have a shorter duration of invasive mechanical ventilation, ECMO course, and ICU stay, as well as fewer infectious complications, and high hospital survival. While it is recognized that an observational study does not imply causality, the favorable outcomes observed in the present study could set the stage for further research and stimulate high-volume ECMO centers to consider in selected patients with severe ARDS the strategy of extubation during ECMO as an integral part of their treatment.

### Supplementary Information

**Additional file 1. Table S1.** Ventilatory, gas exchange, and extracorporeal membrane oxygenation (ECMO) parameters before and at ECMO Day 1. **Table S2.** Ventilatory, gas exchange, and extracorporeal membrane oxygenation (ECMO) parameters at liberation from invasive mechanical ventilation and at ECMO decannulation** Table S3. **Platelet count and coagulation parameters. **Table S4.** Sample characteristics before and after matching in the CTRL and EXT groups. **Table S5.** Unadjusted and adjusted analysis of the duration of ECMO, IMV and ICU length of stay**. Figure S1:** Kaplan–Meier analysis for all-cause mortality stratified by group (EXT vs CTRL) before (A) and after (B) Propensity-score Matching.

## Data Availability

The datasets used and/or analyzed during the current study are available from the corresponding author on reasonable request.

## References

[1] Broman LM, Malfertheiner MV, Montisci A, Pappalardo F (2018). Weaning from veno-venous extracorporeal membrane oxygenation: how I do it. J Thorac Dis.

[2] Swol J, Shekar K, Protti A (2021). Extubate before venovenous extracorporeal membranous oxygenation decannulation or decannulate while remaining on the ventilator? The EuroELSO 2019 Weaning Survey. ASAIO J.

[3] Al-Fares AA, Ferguson ND, Ma J (2021). Achieving safe liberation during weaning from VV-ECMO in patients with severe ARDS: the role of tidal volume and inspiratory effort. Chest.

[4] Langer T, Santini A, Bottino N (2016). "Awake" extracorporeal membrane oxygenation (ECMO): pathophysiology, technical considerations, and clinical pioneering. Crit Care.

[5] Vaquer S, de Haro C, Peruga P, Oliva JC, Artigas A (2017). Systematic review and meta-analysis of complications and mortality of veno-venous extracorporeal membrane oxygenation for refractory acute respiratory distress syndrome. Ann Intensive Care.

[6] Seeliger B, Doebler M, Hofmaenner DA (2022). Intracranial hemorrhages on extracorporeal membrane oxygenation: differences between COVID-19 and other viral acute respiratory distress syndrome. Crit Care Med.

[7] Kalbhenn J, Glonnegger H, Buchsel M, Priebe HJ, Zieger B (2022). Acquired von Willebrand syndrome and desmopressin resistance during venovenous extracorporeal membrane oxygenation in patients with COVID-19: a prospective observational study. Crit Care Med.

[8] Gannon WD, Stokes JW, Bloom S (2021). Safety and feasibility of a protocolized daily assessment of readiness for liberation from venovenous extracorporeal membrane oxygenation. Chest.

[9] Bullen EC, Teijeiro-Paradis R, Fan E (2020). How I select which patients with ARDS should be treated with venovenous extracorporeal membrane oxygenation. Chest.

[10] Hoeper MM, Wiesner O, Hadem J (2013). Extracorporeal membrane oxygenation instead of invasive mechanical ventilation in patients with acute respiratory distress syndrome. Intensive Care Med.

[11] Agerstrand C, Abrams D, Bacchetta M, Brodie D. Endotracheal extubation in patients with respiratory failure receiving venovenous ECMO. 2015. https://www.elso.org/Portals/0/Files/ELSO_ExtubationGuidelines_May2015.pdf.

[12] Olsson KM, Simon A, Strueber M (2010). Extracorporeal membrane oxygenation in nonintubated patients as bridge to lung transplantation. Am J Transplant.

[13] Biscotti M, Gannon WD, Agerstrand C (2017). Awake extracorporeal membrane oxygenation as bridge to lung transplantation: a 9-year experience. Ann Thorac Surg.

[14] Nosotti M, Rosso L, Tosi D (2013). Extracorporeal membrane oxygenation with spontaneous breathing as a bridge to lung transplantation. Interact Cardiovasc Thorac Surg.

[15] Crotti S, Bottino N, Spinelli E (2018). Spontaneous breathing during veno-venous extracorporeal membrane oxygenation. J Thorac Dis.

[16] Crotti S, Bottino N, Ruggeri GM (2017). Spontaneous breathing during extracorporeal membrane oxygenation in acute respiratory failure. Anesthesiology.

[17] Teijeiro-Paradis R, Gannon WD, Fan E (2022). Complications associated with venovenous extracorporeal membrane oxygenation-what can go wrong?. Crit Care Med.

[18] Barbaro RP, Odetola FO, Kidwell KM (2015). Association of hospital-level volume of extracorporeal membrane oxygenation cases and mortality. Analysis of the extracorporeal life support organization registry. Am J Respir Crit Care Med.

[19] Riera J, Alcantara S, Bonilla C (2022). Risk factors for mortality in patients with COVID-19 needing extracorporeal respiratory support. Eur Respir J.

[20] Tukacs M, Cato KD (2021). Extubation during extracorporeal membrane oxygenation in adults: an international qualitative study on experts' opinions. Heart Lung.

[21] Winnersbach P, Rossaint J, Buhl EM (2022). Platelet count reduction during in vitro membrane oxygenation affects platelet activation, neutrophil extracellular trap formation and clot stability, but does not prevent clotting. Perfusion.

[22] Hu W, Zhang J, Wang M (2021). Clinical features and risk factors analysis for hemorrhage in adults on ECMO. Front Med (Lausanne).

[23] MacLaren G, Schlapbach LJ, Aiken AM (2020). Nosocomial infections during extracorporeal membrane oxygenation in neonatal, pediatric, and adult patients: a comprehensive narrative review. Pediatr Crit Care Med.

[24] Grasselli G, Scaravilli V, Di Bella S (2017). Nosocomial infections during extracorporeal membrane oxygenation: incidence, etiology, and impact on patients' outcome. Crit Care Med.

[25] Wu D, Wu C, Zhang S, Zhong Y (2019). Risk factors of ventilator-associated pneumonia in critically iii patients. Front Pharmacol.

[26] Jeon CY, Neidell M, Jia H, Sinisi M, Larson E (2012). On the role of length of stay in healthcare-associated bloodstream infection. Infect Control Hosp Epidemiol.

[27] Bataillard A, Hebrard A, Gaide-Chevronnay L (2017). Extubation in patients undergoing extracorporeal life support. Int J Artif Organs.

[28] Ellouze O, Lamirel J, Perrot J (2019). Extubation of patients undergoing extracorporeal life support. A retrospective study. Perfusion.

[29] Wiesner O, Hadem J, Sommer W, Kuhn C, Welte T, Hoeper MM (2012). Extracorporeal membrane oxygenation in a nonintubated patient with acute respiratory distress syndrome. Eur Respir J.

[30] Henderson WR, Chen L, Amato MBP, Brochard LJ (2017). Fifty years of research in ARDS. Respiratory mechanics in acute respiratory distress syndrome. Am J Respir Crit Care Med.

[31] Schmidt M, de Chambrun MP, Lebreton G (2021). Extracorporeal membrane oxygenation instead of invasive mechanical ventilation in a patient with severe COVID-19-associated acute respiratory distress syndrome. Am J Respir Crit Care Med.

[32] Al-Mumin A, Tarakemeh H, Buabbas S (2022). Liberation from mechanical ventilation before decannulation from venovenous extracorporeal life support in severe COVID-19 acute respiratory distress syndrome. ASAIO J.

[33] Becher T, Schadler D, Rostalski P, Zick G, Frerichs I, Weiler N (2018). Determination of respiratory system compliance during pressure support ventilation by small variations of pressure support. J Clin Monit Comput.

[34] Azarian R, Lofaso F, Zerah F (1993). Assessment of the respiratory compliance in awake subjects using pressure support. Eur Respir J.

[35] Xia J, Gu S, Li M (2019). Spontaneous breathing in patients with severe acute respiratory distress syndrome receiving prolonged extracorporeal membrane oxygenation. BMC Pulm Med.

[36] Thille AW, Muller G, Gacouin A (2019). Effect of postextubation high-flow nasal oxygen with noninvasive ventilation vs high-flow nasal oxygen alone on reintubation among patients at high risk of extubation failure: a randomized clinical trial. JAMA.

[37] Hermens JA, Braithwaite SA, Platenkamp M (2017). Awake ECMO on the move to lung transplantation: serial monitoring of physical condition. Intensive Care Med.

[38] Stahl K, Schenk H, Kuhn C, Wiesner O, Hoeper MM, David S (2021). Extracorporeal membrane oxygenation in non-intubated immunocompromised patients. Crit Care.

[39] Mang S, Reyher C, Mutlak H (2022). Awake extracorporeal membrane oxygenation for COVID-19-induced acute respiratory distress syndrome. Am J Respir Crit Care Med.

[40] Franklin JM, Eddings W, Glynn RJ, Schneeweiss S (2015). Regularized regression versus the high-dimensional propensity score for confounding adjustment in secondary database analyses. Am J Epidemiol.

