# Supplementary material for: Standardized approach for extubation during extracorporeal membrane oxygenation in severe acute respiratory distress syndrome: a prospective observational study
Source: Ann Intensive Care. 2023 Sep 18;13:86. doi: 10.1186/s13613-023-01185-y (PMC10506998; doi:10.1186/s13613-023-01185-y)
Supplement: Supplementary file 1 — Additional file 1. Table S1. Ventilatory, gas exchange, and extracorporeal membrane oxygenation (ECMO) parameters before and at ECMO Day 1. Table S2. Ventilatory, gas exchange, and extracorporeal membrane oxygenation (ECMO) parameters at liberation from invasive mechanical ventilation and at ECMO decannulation Table S3. Platelet count and coagulation parameters. Table S4. Sample characteristics before and after matching in the CTRL and EXT groups. Table S5. Unadjusted and adjusted analysis of the duration of ECMO, IMV and ICU length of stay. Figure S1: Kaplan–Meier analysis for all-cause mortality stratified by group (EXT vs CTRL) before (A) and after (B) Propensity-score Matching. [file 13613_2023_1185_MOESM1_ESM.docx]

**Additional Data**

**A standardized approach for extubation during extracorporeal membrane oxygenation in severe acute respiratory distress syndrome.**

**ECMO case volume, clinical criteria for ECMO consideration, and ECMO technical considerations**

São João University Hospital Center is an 1100-bed tertiary hospital and has the sole ECMO reference center in the north of Portugal, a region with approximately 4 million inhabitants. With a current case volume of ~100 patients/year (~50% VV-ECMO; neonatal and pediatric ECMO representing 5-10% of the total), the ECMO Reference Center is an ELSO member (Center 227). Alvaro Cunqueiro University Hospital is an 845-bed tertiary hospital and has an ECMO reference center for the Galicia region of Spain, a region with approximately 3 million inhabitants. With a current case volume of ~40 patients/year (~70% VV-ECMO), the ECMO Reference Center is an ELSO member (Center 774). Specific ECMO data were collected and presented from dedicated databases. The two ECMO centers participating in the study have developed a close collaboration both at the clinical level through exchanges in carrying out various clinical internships, and at the scientific level through joint participation in several studies on the ECMO field.

Consideration of ECMO in ARDS was based on the presence of severe respiratory failure (Murray score ≥ 3.0 and/or pH ≤ 7.20 under protective invasive mechanical ventilation) with sustained clinical deterioration despite optimal conventional treatment (including prone position unless contraindicated). Aggressive mechanical ventilation (plateau pressure (PPlat) ≥ 30 cmH_2_O and/or fraction of inspired oxygen (FiO_2_) ≥ 0.8) for more than 7 days, severe co-morbidity, and modified Rankin score ≥ 3 were used as contraindications for ECMO.

The ECMO circuits used were heparin-coated and consisted of two cannulae, a centrifugal pump, and a membrane oxygenator with 3/8" connecting tubes (e.g., HLS Set Advanced 7.0 from Maquet-Cardiopulmonary-AG; Hirrlingen, Germany). All cannulation procedures were performed percutaneously by dedicated intensive care medicine specialists from the ECMO center using the Seldinger technique at the bedside in an Intensive Care Unit (ICU). The veno-venous (VV) ECMO configuration was used, with a femoral vein being cannulated with a 25-Fr to 29-Fr cannula for blood outflow from the inferior vena cava. For reinfusion, a 15-cm-long 19-Fr cannula was used, implanted in the right internal jugular vein - femoro-jugular configuration. When the femoro-femoral configuration was used, a 55-cm-long 21-Fr cannula was implanted in the contralateral femoral vein for blood reinfusion. Pure oxygen was used as the sweep gas with a flow from 1 to 12 L/min. Systemic anticoagulation was maintained using unfractionated heparin to a partial thromboplastin time (aPTT) of 1.5 normal. A heat exchanger was used to maintain normothermia. All patients had invasive arterial pressure monitoring and intensivists with two-dimensional echocardiography and pulsed Doppler training were available 24/7.

VVA ECMO configuration was considered for circulatory support in the setting of simultaneous severe respiratory failure and cardiovascular collapse. This consisted in the addition of a 15-cm-long 15-Fr to 19-Fr arterial return cannula to the circuit via a common femoral artery with an anterograde distal perfusion 7-Fr catheter in the ipsilateral superficial femoral artery to prevent limb ischemia. The arterial cannula was then connected to the existing ECMO circuit with a Y-connector from the return limb of the circuit. To regulate flow between the two return cannulae, Hoffman clamps were used on the tubing between the Y-connector and the venous return cannula.

After cannulation, ECMO was optimized to minimize further ventilator-induced lung injury. Regarding oxygenation, ECMO blood flow was maximized to reduce FiO_2_ below 0.6 and maintain hemoglobin saturation above 85%. Positive end-expiratory pressure (PEEP) was maintained > 10 cmH_2_O to avoid lung atelectasis unless contraindicated. The threshold for prophylactic platelet transfusion was 30.000/μL, whereas the targeted post-transfusion goal was 100.000/μL in the presence of active bleeding. Regarding CO_2_ removal, sweep gas flow was progressively increased to achieve a normal pH, small tidal volumes (≤ 6 ml/kg/predicted body weight), and driving pressures below 14 cmH_2_O. Whenever severe respiratory acidosis was present, PaCO_2_ was reduced slowly during the first 24-48h after ECMO initiation to avoid neurologic complications.

At the end of the ECMO run, the extracorporeal system was removed after percutaneous venous decannulation with skin suture. In cases of VVA ECMO, arterial decannulation was performed using the Perclose ProGlide (Abbott Vascular, Chicago, IL) closure device. An eco-Doppler was routinely performed to exclude deep vein thrombosis and arterial pseudoaneurysm in the previously cannulated vessels.

**Statistical analysis**

Categorical variables are described as frequencies (proportions) and continuous variables are expressed as median (interquartile range). Comparisons between groups (EXT vs. CTRL) were performed using the Mann-Whitney U test for continuous variables, while the Chi-Square Test was used for categorical variables. An unadjusted comparison between groups was carried out for pre-ECMO variables, clinical course and in ICU complications, and outcome measures (*Tables 1, 2, 4* and *Supplemental Tables 1, 2, and 3*). A multivariate logistic regression model was built using a stepwise approach to identify relevant associations with group assignment (*Table 2*). The clinical endpoints analysed were all-cause death, ventilator-associated pneumonia (VAP), nosocomial infection, and tracheostomy. Standard and least absolute shrinkage and selection operator (LASSO) logistic regression models were used to assess the association between groups and the clinical endpoints, adjusting for relevant covariates. LASSO is a penalized regression approach that estimates the regression coefficients by maximizing the log-likelihood function (or the sum of squared residuals) with a constraint on the sum of the absolute values of the regression coefficients, enhancing the prediction accuracy and interpretability in high-dimensional models.^40^ Standard logistic regression was adjusted for key mechanistic and biological covariates, namely age, sex, hypertension, SAPS II, SOFA, pre-ECMO FiO2 and log-transformed lactate, and log-transformed ICU length of stay (LOS). LASSO logistic regression was adjusted for: i) continuous variables (age, SAPS II, SOFA, hospital-to-ECMO and IMV-to-ECMO times, pre-ECMO FiO2 and lactate, ICU LOS and ECMO duration); ii) categorical variables (sex, hypertension, obesity, dyslipidemia, smoking, diabetes, alcoholism, ARDS type and ARDS etiology); and iii) interaction between all continuous and categorical variables (*Figure 2*). To further account for measured confounding, we then performed a non-parsimonious 1:1 nearest-neighbor propensity score (PS) matching without replacement based on logistic regression including 12 clinically relevant independent covariates, regardless of differences between groups (*Supplemental Table 4*). Covariate balance was assessed by standardized bias. Linear regression was used to assess the impact of group assignment on ECMO duration, IMV duration, and LOS times, adjusting for clinically relevant covariates (*Supplemental Table 5*). Kaplan-Meier curve analysis, before (Panel A) and after (Panel B) PS matching (*Supplemental Figure 1*), was performed using the log-rank test. All statistical analyses were performed with Stata® (StataCorp. 2021. Stata Statistical Software: Release 17.0. College Station, TX: StataCorp LLC).

**Table S1:** Ventilatory, gas exchange, and extracorporeal membrane oxygenation (ECMO) parameters before and at ECMO Day 1.

|  | All  (n=254) | CTRL  (n=200) | EXT  (n=54) | *P-value* |
| --- | --- | --- | --- | --- |
| Pre-ECMO |  |  |  |  |
| FiO_2_ (%) | 100 (75-100) | 100 (80-100) | 80 (70-100) | **0.02** |
| PEEP (cmH_2_O) | 12 (10-14) | 12 (9.5-14) | 12 (10-14) | 0.36 |
| Tidal volume / PBW (mL/kg) | 7.3 (6.7-8.2) | 7.5 (6.7-8.2) | 7.1 (6.5-7.6) | 0.22 |
| Minute ventilation (L/min) | 12.0 (9.9-14.0) | 12.3 (9.9-14.3) | 10.8 (9.0-13.2) | 0.08 |
| Plateau pressure (cmH_2_O) | 27 (24-30) | 28 (24-30) | 26 (24-29) | 0.16 |
| Static RS compliance (mL/cmH_2_O) | 25 (18-35) | 25 (18-35) | 28 (20-35) | **0.02** |
| PaO_2_/FiO_2_ (mmHg) | 86 (68-100) | 85 (66-100) | 92 (72-100) | 0.22 |
| PaCO_2_ (mmHg) | 55 (47-65) | 56 (47-66) | 54 (45-64) | 0.20 |
| pH | 7.35 (7.26-7.44) | 7.35 (7.26-7.45) | 7.34 (7.26-7.42) | 0.24 |
| Lactate (mM) | 1.5 (1.1-2.4) | 1.6 (1.1-2.6) | 1.3 (1.0-1.6) | **0.01** |
| ECMO Day 1 |  |  |  |  |
| FiO_2_ (%) | 40 (40-60) | 50 (40-60) | 40 (35-50) | **<0.01** |
| PEEP (cmH_2_O) | 10 (9-12) | 10 (9-12) | 10 (10-12) | 0.50 |
| Tidal volume / PBW (mL/kg) | 4.8 (3.3-5.7) | 4.8 (3.2-5.9) | 5.1 (4.0-5.6) | 0.45 |
| Minute ventilation (L/min) | 4.0 (2.6-8.0) | 4.0 (2.5-9.9) | 3.8 (2.8-5.0) | 0.13 |
| Plateau pressure (cmH_2_O) | 22 (20-24) | 22 (20-24) | 22 (19-23) | 0.31 |
| Static RS compliance (mL/cmH_2_O) | 25 (18-35) | 25 (18-35) | 28 (20-35) | 0.53 |
| PaO_2_/FiO_2_ (mmHg) | 211 (173-270) | 208 (171-270) | 223 (190-275) | 0.25 |
| PaCO_2_ (mmHg) | 46 (41-51) | 46 (41-52) | 48 (41-50) | 0.67 |
| pH | 7.39 (7.33-7.45) | 7.39 (7.34-7.46) | 7.37 (7.33-7.42) | 0.11 |
| Lactate (mM) | 1.5 (1.1-2.0) | 1.6 (1.1-2.3) | 1.3 (1.1-1.7) | 0.06 |
| ECMO blood flow (L/min) | 4.2 (4.0-4.7) | 4.1 (4.0-4.6) | 4.3 (4.0-4.7) | 0.48 |
| ECMO sweep (L/min) | 4.0 (3.0-5.0) | 4.0 (3.0-5.0) | 3.5 (3.0-5.0) | 0.08 |

Data is presented as median (interquartile range). CTRL, group without extubation during ECMO; ECMO, extracorporeal membrane oxygenation; EXT, group with extubation during ECMO; FiO_2_, fraction of inspired oxygen; PaO_2_, partial pressure of oxygen in arterial blood; PaCO_2_, partial pressure of carbon dioxide in arterial blood; PBW, predicted body weight; PEEP, positive end-expiratory pressure; RS, respiratory system; Sweep, ECMO sweep gas flow.

**Table S2:** Ventilatory, gas exchange, and extracorporeal membrane oxygenation (ECMO) parameters at liberation from invasive mechanical ventilation and at ECMO decannulation.

|  | All  (n=254) | CTRL  (n=200) | EXT  (n=54) | *P-value* |
| --- | --- | --- | --- | --- |
| Liberation From IMV Day |  |  |  |  |
| FiO_2_ (%) | 35 (30-40) | 35 (28-40) | 35 (30-40) | 0.13 |
| PEEP (cmH_2_O) | 8 (6-10) | 7 (5-8) | 8 (7-10) | **<0.01** |
| Tidal volume / PBW (mL/kg) | 7.1 (5.7-8.2) | 7.8 (7.1-8.8) | 5.6 (4.8-6.4) | **<0.01** |
| Minute ventilation (L/min) | 7.2 (3.6-10.0) | 8.8 (6.7-13.3) | 4.9 (3.2-5.6) | **<0.01** |
| Plateau pressure (cmH_2_O) | 19 (16-21) | 18 (16-21) | 20 (16-22) | 0.34 |
| Static RS compliance (mL/cmH_2_O) | 38 (29-50) | 45 (35-57) | 29 (17-38) | **<0.01** |
| PaO_2_/FiO_2_ (mmHg) | 294 (240-353) | 303 (250-371) | 254 (212-313) | **<0.01** |
| PaCO_2_ (mmHg) | 40 (37-44) | 39 (36-43) | 42 (39-45) | **<0.01** |
| pH | 7.46 (7.42-7.49) | 7.48 (7.44-7.55) | 7.43 (7.39-7.46) | **<0.01** |
| Lactate (mM) | 1.1 (0.77-1.6) | 1.1 (0.75-1.9) | 1.1 (0.79-1.4) | 0.33 |
| ECMO blood flow (L/min) | - | - | 4.1 (3.8-4.5) | - |
| ECMO sweep (L/min) | - | - | 4.0 (3.0-5.0) | - |
| ECMO Decannulation Day |  |  |  |  |
| FiO_2_ (%) | 35 (30-40) | 35 (30-40) | 40 (32-40) | 0.31 |
| PEEP (cmH_2_O) | - | 8 (7-10) | - | - |
| Tidal volume / PBW (mL/kg) | - | 7.9 (7.1-9.5) | - | - |
| Minute ventilation (L/min) | - | 9.5 (8.0-12.0) | - | - |
| Plateau pressure (cmH_2_O) | - | 21 (18-22) | - | - |
| Static RS compliance (mL/cmH_2_O) | - | 42 (32-57) | - | - |
| PaO_2_/FiO_2_ (mmHg) | 293 (234-360) | 292 (245-362) | 298 (223-325) | 0.30 |
| PaCO_2_ (mmHg) | 42 (38-46) | 43 (39-47) | 38 (35-41) | **<0.01** |
| pH | 7.44 (7.41-7.48) | 7.44 (7.40-7.49) | 7.45 (7.43-7.47) | 0.34 |
| Lactate (mM) | 1.0 (0.73-1.2) | 1.0 (0.76-1.3) | 0.94 (0.70-1.1) | 0.12 |
| ECMO blood flow (L/min) | 3.8 (3.5-4.1) | 3.9 (3.5-4.2) | 3.6 (3.4-3.9) | **0.13** |
| ECMO sweep (L/min) | 3.0 (1.0-5.0) | 2.0 (1.0-5.0) | 1.0 (1.0-2.0) | **0.04** |

Data is presented as median (interquartile range). CTRL, group without extubation during ECMO; ECMO, extracorporeal membrane oxygenation; EXT, group with extubation during ECMO; FiO_2_, fraction of inspired oxygen; PaO_2_, partial pressure of oxygen in arterial blood; PaCO_2_, partial pressure of carbon dioxide in arterial blood; PBW, predicted body weight; PEEP, positive end-expiratory pressure; RS, respiratory system; Sweep, ECMO sweep gas flow.

**Table S3:** Platelet count and coagulation parameters.

|  | All  (n=254) | CTRL  (n=200) | EXT  (n=54) | *P-value* |
| --- | --- | --- | --- | --- |
| Pre-ECMO |  |  |  |  |
| Platelets (10^9^/L) | 198 (132-265) | 182 (104-258) | 215 (161-294) | **0.04** |
| aPTT (sec.) | 42 (35-52) | 44 (36-54) | 38 (33-47) | **0.02** |
| PT (sec.) | 14 (12-15) | 14 (13-15) | 12 (11-13) | **<0.01** |
| D-dimers (µg/dL) | 4.3 (2.7-9.3) | 4.0 (2.5-11.3) | 5.1 (3.0-8.4) | 0.29 |
| Fibrinogen (mg/dL) | 562 (435-700) | 553 (432-700) | 584 (448-679) | 0.35 |
| ECMO Day 1 |  |  |  |  |
| Platelets (10^9^/L) | 195 (119-261) | 174 (103-248) | 228 (150-263) | **0.04** |
| aPTT (sec.) | 44 (37-50) | 44 (39-50) | 42 (35-50) | 0.30 |
| PT (sec.) | 14 (13-16) | 14 (13-15) | 13 (12-17) | 0.43 |
| D-dimers (µg/dL) | 4.9 (2.4-11.8) | 4.7 (2.2-12.2) | 5.7 (3.0-10.7) | 0.33 |
| Fibrinogen (mg/dL) | 529 (404-700) | 528 (387-703) | 530 (453-648) | 0.66 |
| ECMO Day 3 |  |  |  |  |
| Platelets (10^9^/L) | 189 (104-238) | 167 (89-223) | 212 (144-249) | **<0.01** |
| aPTT (sec.) | 48 (43-55) | 48 (43-56) | 48 (44-55) | 0.93 |
| PT (sec.) | 14 (12-15) | 14 (12-15) | 13 (13-15) | 0.84 |
| D-dimers (µg/dL) | 6.1 (2.7-15.9) | 5.9 (2.6-16.2) | 6.1 (2.8-14.3) | 0.96 |
| Fibrinogen (mg/dL) | 516 (390-653) | 510 (387-684) | 521 (405-601) | 0.96 |
| ECMO Day 7 |  |  |  |  |
| Platelets (10^9^/L) | 173 (126-245) | 165 (119-224) | 213 (164-335) | **<0.01** |
| aPTT (sec.) | 50 (45-57) | 51 (44-57) | 50 (45-55) | 0.65 |
| PT (sec.) | 13 (12-14) | 13 (12-14) | 14 (12-18) | 0.21 |
| D-dimers (µg/dL) | 9.7 (3.9-17.8) | 9.3 (3.8-18.9) | 9.8 (5.6-15.1) | 0.85 |
| Fibrinogen (mg/dL) | 517 (410-641) | 515 (408-652) | 522 (416-635) | 0.88 |

Data are presented as median (interquartile range). APTT, activated partial thromboplastin time; CTRL, group without extubation during ECMO; ECMO, extracorporeal membrane oxygenation; EXT, group with extubation during ECMO; PT, Prothrombin Time; Sec., seconds.

**Table S4.** Sample characteristics before and after matching in the CTRL and EXT groups.


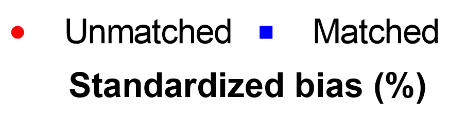


| **Variables** | **Unmatched** | | |  | **Matched** | | |  |
| --- | --- | --- | --- | --- | --- | --- | --- | --- |
|  | **CTRL**  (n=200) | **EXT**  (n=54) | **P-value** |  | **CTRL**  (n=54) | **EXT**  (n=54) | **P-value** |  |
| **Age (*years*)**, median (IQR) | 52 (42-60) | 50 (41-58) | 0.29 |  | 51 (42-59) | 50 (41-58) | 0.76 | 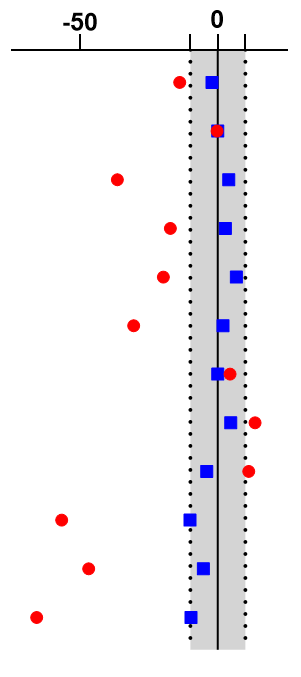 |
| **Male Gender**, n (%) | 141 (70.5) | 38 (70.4) | 0.98 |  | 38 (70.4) | 38 (70.4) | 1 |  |
| **Hypertension**, n (%) | 82 (41.0) | 13 (24.1) | **0.023** |  | 12 (22.2) | 13 (24.1) | 0.82 |  |
| **ARDS Type (*Pulmonary*)**, n (%) | 176 (88.9) | 51 (94.4) | 0.23 |  | 52 (96.3) | 51 (94.4) | 0.65 |  |
| **Hospital to ECMO (*days*),** median (IQR) | 4 (1-8) | 4 (1-7) | 0.33 |  | 4 (2-6) | 4 (1-7) | 0.72 |  |
| **IMV to ECMO (*days*),** median (IQR) | 3 (1-6) | 2 (1-5) | 0.07 |  | 2 (1-3) | 2 (1-5) | 0.71 |  |
| **Pre-ECMO PP**, n (%) | 154 (77.8) | 45 (83.3) | 0.41 |  | 44 (81.5) | 45 (83.3) | 0.8 |  |
| **Pre-ECMO NMB**, n (%) | 193 (97.5) | 52 (98.1) | 0.79 |  | 53 (98.1) | 53 (98.1) | 1 |  |
| **ECMO Retrieval**, n (%) | 134 (67.0) | 39 (72.2) | 0.46 |  | 40 (74.1) | 39 (72.2) | 0.83 |  |
| **SAPS II**, median (IQR) | 49 (36-60) | 39 (24-54) | **<0.001** |  | 42 (29-52) | 39 (24-54) | 0.44 |  |
| **SOFA**, median (IQR) | 8 (6-12) | 7 (4-9) | **0.005** |  | 7 (5-8) | 7 (4-9) | 0.84 |  |
| **ICU LOS (*days*),** median (IQR) | 37 (22-63) | 19 (13-32) | **<0.001** |  | 27 (15-37) | 19 (13-32) | 0.13 |  |

Percentage standardized bias and an absolute 10% interval cut-off used to assess balance between covariates are represented in the rightmost column. ARDS, Acute respiratory distress syndrome; IMV, invasive mechanical ventiation; PP, prone position; NMB, neuromuscular blocking agents; SAPS II, Simplified Acute Physiology Score II at ICU admission; SOFA, Sequential Organ Failure Assessment score at ECMO cannulation; LOS: length of stay; IQR: interquartile range.

**Table S5.** Unadjusted and adjusted analysis of the duration of ECMO, IMV and ICU length of stay

| **Outcomes^1^** |  | |  | |
| --- | --- | --- | --- | --- |
|  | **Unadjusted** | ***P-value*** | **Adjusted^2^** | ***P-value*** |
| **ECMO Duration** |  |  |  |  |
| **CTRL** | *Reference* |  | *Reference* |  |
| **EXT** | -9.6 (-18.5, -0.7) | **0.036** | -14.4 (-23.6, -5.2) | **0.002** |
| **IMV Duration** |  |  |  |  |
| **CTRL** | *Reference* |  | *Reference* |  |
| **EXT** | -28.1 (-37.6, -18.5) | **<0.001** | -34.6 (-44.4, -24.8) | **<0.001** |
| **ICU LOS** |  |  |  |  |
| **CTRL** | *Reference* |  | *Reference* |  |
| **EXT** | -21.2 (-31.6, -10.7) | **<0.001** | -28.4 (-39.2, -17.7) | **<0.001** |

^1^ Linear regression coefficients are presented with their 95% confidence interval.

^2^ Adjusted for age, sex, hypertension, pre-ECMO FiO2, lactate, neuromuscular blockade and prone position, hospital-to-ECMO and IMV-to-ECMO times, ECMO retrieval, SAPSII and SOFA

**Figure S1:** Kaplan-Meier analysis for all-cause mortality stratified by group (EXT vs CTRL) before (A) and after (B) Propensity-score Matching.


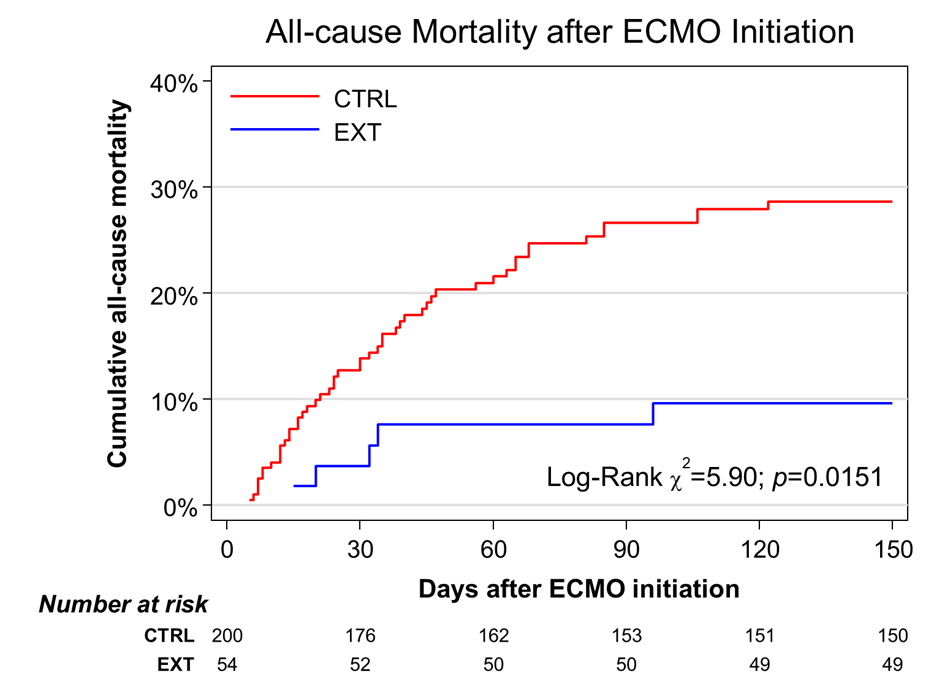


**A**


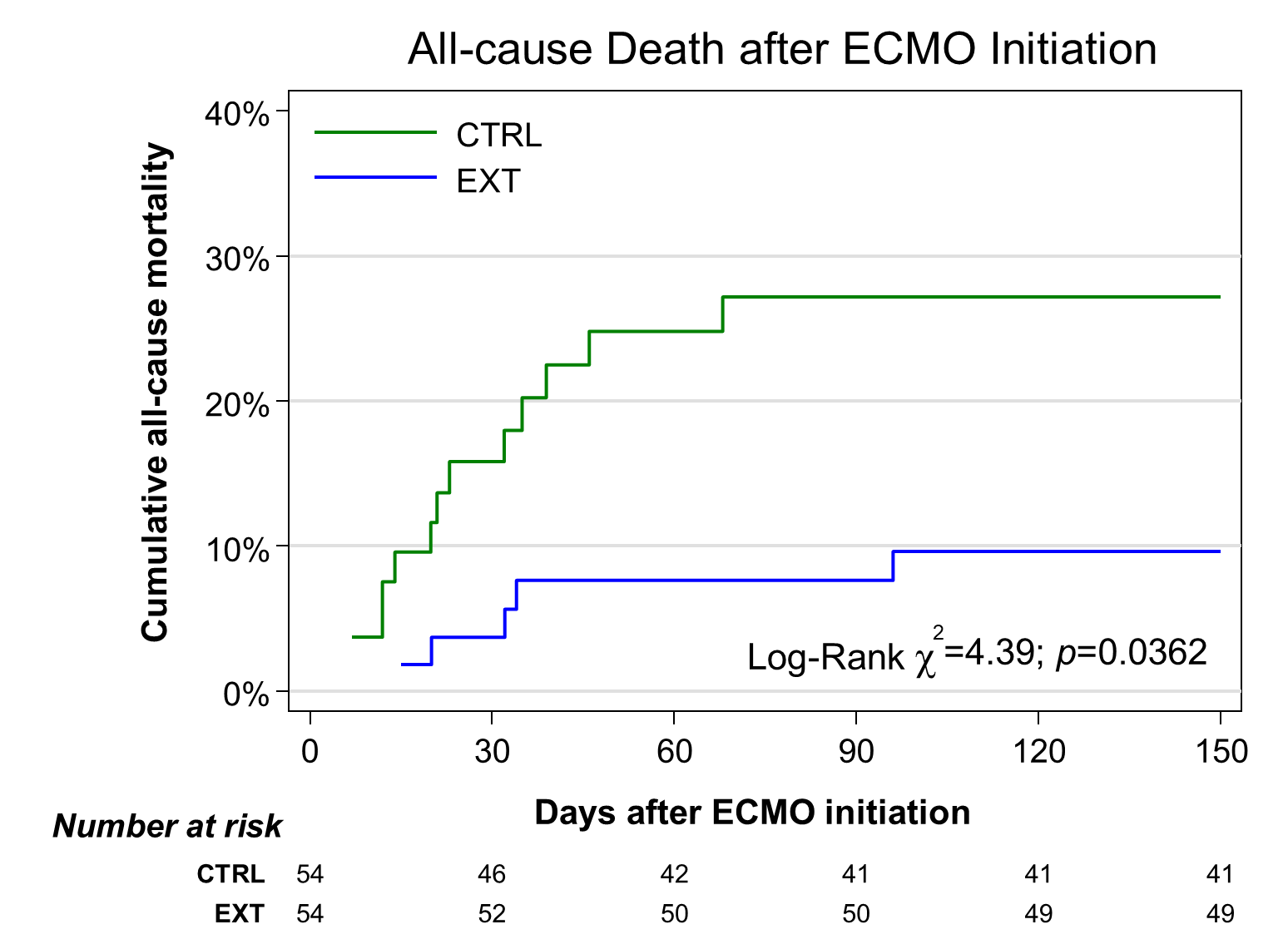


**B**
